# Allogeneic skin transplantation induces transient fibrosis that undergoes spontaneous regression in newts

**DOI:** 10.1186/s41232-026-00438-0

**Published:** 2026-08-01

**Authors:** Kento Hosomi, Maya Nagata, Mika Okada, Kodai Hirano, Eishin Yamada, Toshinori Nakagawa, Kazuaki Maruyama, Chihena Hansini Banda, Makoto Shiraishi, Kanako Danno, Chikafumi Chiba, Mitsunaga Narushima

**Affiliations:** 1 Department of Plastic and Reconstructive Surgery, Graduate School of Medicine, Mie University, Mie, Japan; 2 Independent Researcher, Otsu, Shiga Japan; 3 Department of Pathology and Matrix Biology, Graduate School of Medicine, Mie University, Mie, Japan; 4 Plastic and Reconstructive Surgery Unit, Department of Surgery, The University Teaching Hospital, Lusaka, Zambia; 5 Department of Plastic and Reconstructive Surgery, The University of Tokyo Hospital, Tokyo, Japan; 6 Faculty of Life and Environmental Sciences, University of Tsukuba, Tsukuba, Japan

**Keywords:** Fibrosis regression, Allogeneic skin grafting, Macrophages, Immune response, Newt, Regenerative wound healing

## Abstract

**Background:**

In humans, large skin defects require tissue transplantation because adult skin lacks full regenerative capacity. However, allogeneic grafts are typically rejected due to strong immune responses, whereas autologous grafts often result in severe fibrosis and permanent scarring. In contrast, regenerative vertebrates such as newts can restore tissues with minimal scarring, suggesting the presence of distinct mechanisms regulating tissue repair. How such organisms respond to immune activation induced by non-self tissue transplantation remains unclear. In this study, we aimed to determine how adult newts respond to allogeneic skin transplantation and to examine whether fibrosis induced under such conditions is sustained or resolved.

**Methods:**

We established an allogeneic skin graft model in adult Japanese fire-bellied newts and compared it with autologous grafting. Graft fate, tissue morphology, fibrosis, and immune cell dynamics were analyzed histologically over time. To assess the contribution of macrophage-lineage cells, animals were treated with clodronate liposomes prior to transplantation.

**Results:**

In contrast to mammals, allogeneic grafts did not exhibit typical features of acute rejection and did not show apparent graft loss. Following transplantation, grafts exhibited a characteristic sequence of changes: initial vascular integration, subsequent loss of detectable perfusion, and progressive remodeling of graft appearance. At the donor–host interface, a distinct collagen-rich fibrotic tissue formed, accompanied by marked leukocyte infiltration. This tissue appeared at 2 weeks, reached maximal thickness at 4 weeks, and regressed by 8 weeks. During this period, the graft surface gradually acquired host-like pigmentation, while structural elements of donor tissue remained detectable histologically. Iba1-positive macrophage-lineage cells accumulated within the graft during the fibrotic phase and decreased as the tissue regressed. Depletion of these cells did not prevent fibrosis formation but delayed its regression and prolonged graft thickening.

**Conclusions:**

Allogeneic skin transplantation in adult newts induces a distinct response in which non-self tissue is not completely lost and a collagen-rich fibrotic tissue forms transiently and subsequently undergoes resolution. These findings suggest that fibrosis induced under non-self immune activation is not necessarily a terminal state but may represent a dynamically regulated process capable of resolution.

**Supplementary Information:**

The online version contains supplementary material available at 10.1186/s41232-026-00438-0.

## Background

Extensive skin tissue injury in humans requires surgical reconstruction using tissue transplantation because humans are unable to fully regenerate skin tissue [1, 2]. Although tissue transplantation can close large tissue defects, allogeneic skin grafts in mammals—transplants from another individual—elicit strong immune rejection and are ultimately rejected and slough off over time [3, 4]. Therefore, in clinical practice, autologous tissue from the patient is used as the donor site to achieve wound closure [1, 5]. While autologous skin grafting successfully closes wounds, it is inevitably accompanied by fibrotic remodeling and scar formation at the grafted wound site, resulting in permanent scars that cause aesthetic and functional impairments [6, 7]. The processes of graft engraftment and wound healing are deeply associated with immune responses [8–10]. Even in autologous grafts, immune responses promote fibrosis, whereas in allogeneic grafts, the transplanted tissue is eventually rejected and sloughs off, preventing complete and functional closure of extensive tissue defects [3, 5].

In contrast, regenerative vertebrates such as newts are capable of restoring complex tissues without leaving significant fibrosis [11–13]. This process is thought to involve unique immunological properties that suppress excessive immune responses [14–16]. How do newts respond to transplanted tissues in order to achieve scarless repair? Do they prevent fibrosis even under conditions of strong immune activation, such as in response to non-self tissue transplantation?

Allogeneic transplantation provides a useful model in which non-self tissue is grafted onto injured tissue, enabling the observation of repair processes under active immune interactions between the host and graft [17]. In this study, we applied an allogeneic skin graft model to adult newts and analyzed tissue responses accompanying immune activation. Surprisingly, although fibrosis—typically minimal in regenerative species—was induced, it was transient and progressively regressed over time. Furthermore, depletion of clodronate-sensitive immune cells delayed this regression, suggesting that these cells may contribute to active fibrosis regression in newts.

This study aims to elucidate how regenerative organisms respond to strong immune activation induced by allogeneic transplantation. Here, we report a unique phenomenon that provides insight into the type of immune response required for achieving complete and scarless closure of severe tissue injury.

## Methods

### Animals

Wild-type adult Japanese fire-bellied newts (Cynops pyrrhogaster) were obtained from Charm Co., Ltd. (Oura-gun, Gunma, Japan; https://www.charm.co.jp/) and maintained at the Department of Plastic Surgery, Mie University. As wild-caught animals, they were considered genetically unrelated (non-sibling). Adult newts weighing 3–7 g were maintained under standard laboratory conditions in freshwater at 20–24 °C, as described previously [18]. Light–dark cycles were not strictly controlled. Animals were randomly assigned to experimental groups without distinction of sex. After any experimental procedures, newts were reared individually.

All experimental procedures were conducted in accordance with institutional guidelines for animal experimentation approved by the relevant Animal Care and Use Committees.

### Anesthesia

Animals were anesthetized with 0.1% FA100 solution (4-allyl-2-methoxyphenol; DS Pharma Animal Health, Osaka, Japan) dissolved in water at room temperature for 45 min to 1 h before experimental procedures.

### Allogeneic or autologous skin transplantation

Following anesthesia, an approximately 3 × 3 mm ventral skin graft was harvested, and a skin defect of the same size was created on the dorsal surface. The graft was rotated by 90° and transplanted onto the defect site and secured with eight interrupted 9–0 nylon sutures under a surgical microscope. No ointment or dressing was applied.

The host individuals were defined according to the purpose of the experiment. For the allogeneic skin graft, two newts were grouped and harvested skin was grafted onto another individual. For autologous skin graft, harvested skin was grafted on the dorsal tissue defect of the same individual.

### Histochemistry

Specimens were fixed in 4% paraformaldehyde (PFA; Wako) at 4 °C for 4 h. Fixed samples were washed twice with distilled water and stored in 70% ethanol at 4 °C. Specimens were dehydrated through graded ethanol, cleared with xylene, embedded in paraffin, and sectioned at 6 μm thickness.

Sections were stained with hematoxylin and eosin (HE), Elastica-Sirius Red (ESR), or Masson’s trichrome according to standard protocols.

For immunohistochemistry, sections were deparaffinized and rehydrated through a series of xylene and ethanol. Antigen retrieval was performed by heating sections in citrate buffer (pH 6.0) using a microwave for 10 min, followed by cooling in a water bath for 20 min. Sections were incubated with Blocking One Histo (Nacalai Tesque) for 1 h at room temperature. Sections were incubated with primary antibodies against α-smooth muscle actin (αSMA; Proteintech, #14,395–1-AP, 1:500) or Iba1 (Cell Signaling Technology, #17,198, 1:200) in 4% donkey serum/Can Get Signal Solution 1 (Toyobo) overnight at 4 °C. After washing with 0.04% Tween-20 in PBS, sections were incubated with Alexa Fluor 488–conjugated goat anti-rabbit IgG (H + L) (Invitrogen, #A-11008, 1:1000) in 4% donkey serum/Can Get Signal Solution 2 (Toyobo) for 1 h at room temperature.

After washing with PBS, slides were mounted using Fluoromount (Diagnostic BioSystems) for fluorescence microscopy.

For enzyme-based detection, sections were incubated with secondary antibodies from the Histofine Simple Stain System (Nichirei Biosciences) for 1 h according to the manufacturer’s instructions, and peroxidase activity was visualized using DAB (3,3′-diaminobenzidine)-H₂O₂.

Images were acquired using a Keyence BZ-X700 microscope. Iba1-positive cells and area were quantified in defined regions of interest using ImageJ.

### Histological analysis

Tissue thickness beneath the grafted skin was measured by histomorphometric analysis. Sections were stained with HE. Reactive fibrotic tissue was defined as the region between the undersurface of the grafted skin and the surface of the host muscle. Mean vertical thickness was calculated by measuring the distance between the lower boundary of the grafted skin and the upper surface of the host muscle using ImageJ.

For leukocyte counts, segmented leukocytes were identified based on morphological characteristics defined in peripheral blood after May–Giemsa staining, and subsequently counted on HE-stained sections [19, 20]. The central region of the graft and adjacent regions on both sides were analyzed.

To assess phagocytic uptake and to validate depletion of phagocytic/macrophage-lineage cells, DiO (3,3′-dioctadecyloxacarbocyanine perchlorate)-labeled liposomes (Fluoroliposome® Kit, Encapsula NanoSciences, #SKU CLD-8908) or commercially available India ink (Kuretake, Japan; diluted 1:100 in saline) were intravenously injected into the midline abdominal vein under a surgical microscope. Peripheral blood leukocytes were examined 24 h after injection using May–Giemsa staining.

### Clodronate injections

Clodronate liposomes (Encapsula Nano Sciences) or PBS liposomes (control) were administered intraperitoneally at 5 μl/g body weight at 96, 48, and 24 h prior to surgical procedures. This triple-injection schedule was adopted based on a previously reported macrophage depletion protocol in axolotls [14], with modification of the route of administration for use in adult newts. Macrophage depletion was verified by a reduction in Iba1-positive cells in the spleen.

### Statistical analysis

Comparisons between two independent groups were performed using unpaired two-tailed Welch’s t-tests. Longitudinal data obtained repeatedly from the same animals were analyzed using repeated-measures ANOVA or a mixed-effects model when values were missing. For longitudinal comparisons between treatment groups, two-way repeated-measures ANOVA or a mixed-effects model was used, followed by appropriate multiple-comparison tests. All data are presented as mean ± standard error of the mean (SEM). A *p* value < 0.05 was considered statistically significant. Statistical analyses were conducted using GraphPad Prism 10 (GraphPad Software, San Diego, CA, USA).

## Results

### Allogeneic skin grafts avoid graft loss and undergo progressive changes in gross appearance in newts

**Fig. 1:**
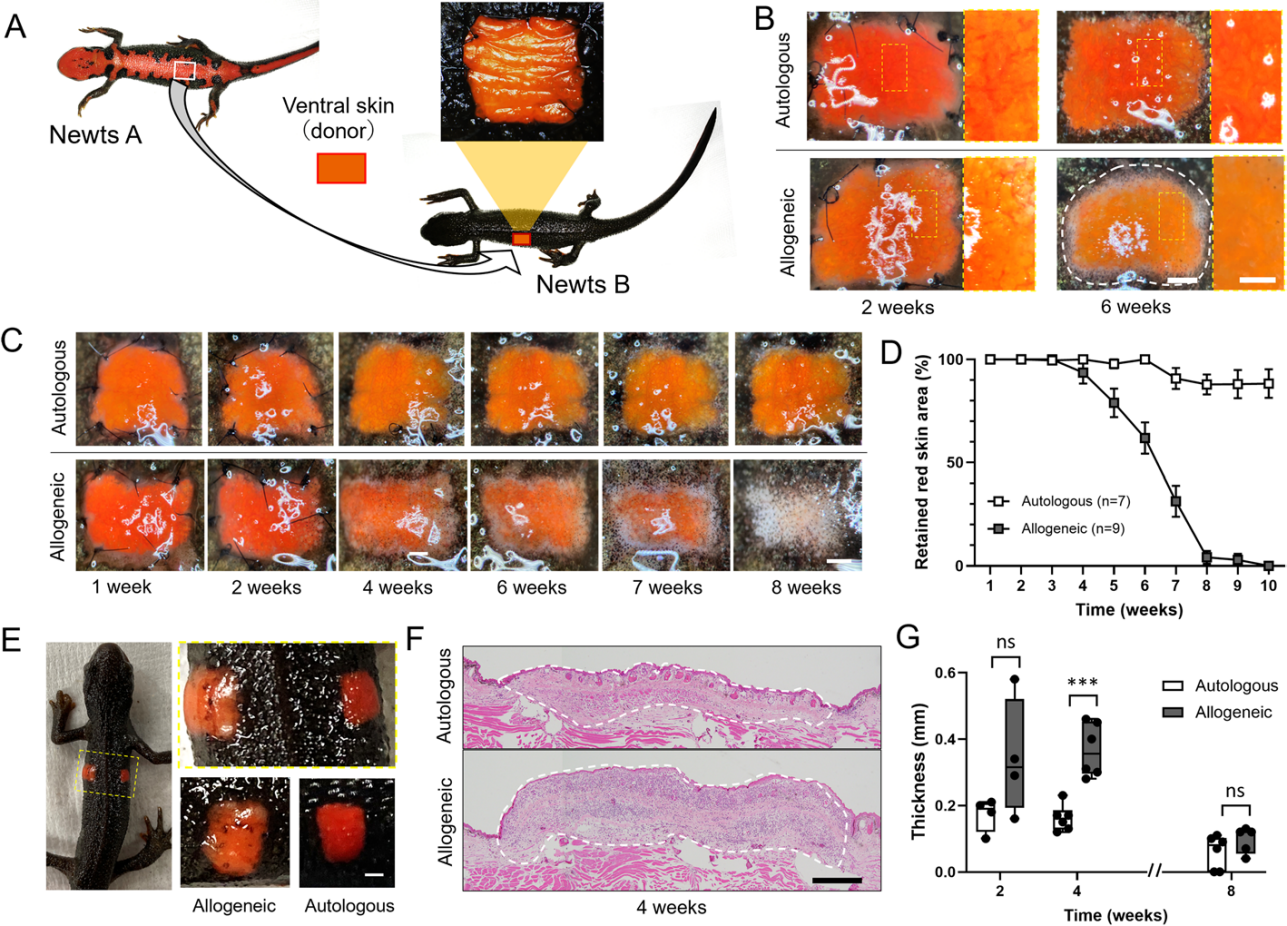
Allogeneic skin grafts avoid graft loss and undergo progressive changes in gross appearance in newts. **A** Schematic of the allogeneic skin transplantation model. Full-thickness dorsal skin defects were created in newts and covered with either autologous or allogeneic ventral skin grafts (3 mm × 3 mm) to visualize graft boundaries. The grafts were secured with eight sutures under a surgical microscope. **B** Representative images at 2 and 6 weeks post-transplantation. At 2 weeks, blood flow was detectable in both autologous and allogeneic graft regions. By 6 weeks, blood flow was no longer detectable in the allogeneic graft region but persisted in autologous grafts. Yellow dashed boxes indicate magnified regions. Scale bars, 500 μm (main) and 100 μm (insets). **C** Time course of graft appearance. Autologous grafts maintained stable morphology and color over time. In contrast, allogeneic grafts showed progressive replacement of the red ventral-skin appearance by surrounding skin-like pigmentation from 6 weeks, with further replacement evident by 8 weeks. Scale bars, 500 μm. **D** Quantification of retained red skin area. Allogeneic grafts showed a progressive decrease in red coloration over time. Data were analyzed by two-way repeated-measures ANOVA (mixed-effects model, REML), revealing a significant interaction between group and time (*p* < 0.0001). **E** Macroscopic appearance at 4 weeks. Allogeneic grafts exhibited marked swelling and thickening compared with autologous grafts. Scale bars, 500 μm. **F** Histological analysis at 4 weeks. Allogeneic grafts showed prominent cellular infiltration and thickening at the donor–host interface. White dashed lines indicate donor boundaries. Scale bars, 500 μm. **G** Quantification of tissue thickness. Allogeneic grafts showed transient thickening, peaking at 4 weeks and regressing by 8 weeks. Data were analyzed using Welch’s t-test. All data are presented as mean ± SEM. *p < 0.05, **p < 0.01, ***p < 0.001, ns: not significant

Unlike mammalians, allogeneic skin grafts in newts did not show apparent graft loss during the observation period. Full-thickness dorsal skin defects were created and covered with allogeneic grafts (Fig. 1A). To enable clear identification of graft boundaries, red abdominal skin was used to cover 3 mm square defects.

The transplanted non-self skin did not undergo complete elimination. At 2 weeks postoperatively, blood flow was detected within the allogeneic graft, comparable to autologous grafts, indicating that non-self tissue can transiently integrate with the host circulation. However, by 6 weeks, blood flow was no longer detectable in allogeneic grafts, whereas it remained evident in autologous grafts (Fig. 1B, Movie 1). This finding suggests that detectable vascular perfusion was reduced or absent in allogeneic grafts by 6 weeks; nevertheless, the graft region remained in place without apparent graft loss.

The subsequent course differed markedly from autologous grafts. In autologous transplantation, graft morphology and color remained stable without invasion from surrounding tissue. In contrast, allogeneic grafts showed progressive replacement of the red ventral-skin-colored appearance by surrounding skin-like pigmentation from 6 weeks onward, and the red color had largely disappeared by 8 weeks (Fig. 1C, D). These findings suggested that the allogeneic graft region underwent progressive gross remodeling rather than simple graft failure.

At 4 weeks postoperatively, swelling and thickening were observed in allogeneic grafts but not in autologous grafts (Fig. 1E). Histological analysis revealed prominent cellular infiltration and the formation of a distinct tissue at the donor–host interface, here termed reactive thickened tissue, characterized by transient thickening and cellular infiltration (Fig. 1F). This thickened tissue could not be clearly distinguished as either donor- or host-derived. It first appeared at 2 weeks, reached maximal thickness at 4 weeks, and regressed by 8 weeks (Fig. 1G).

These results indicate that allogeneic skin grafts in newts do not undergo apparent graft loss but instead show a distinctive process characterized by progressive color replacement, transient tissue thickening, and subsequent remodeling.

### Allogeneic grafts are associated with transient fibrosis and epidermal remodeling without complete graft loss

**Fig. 2:**
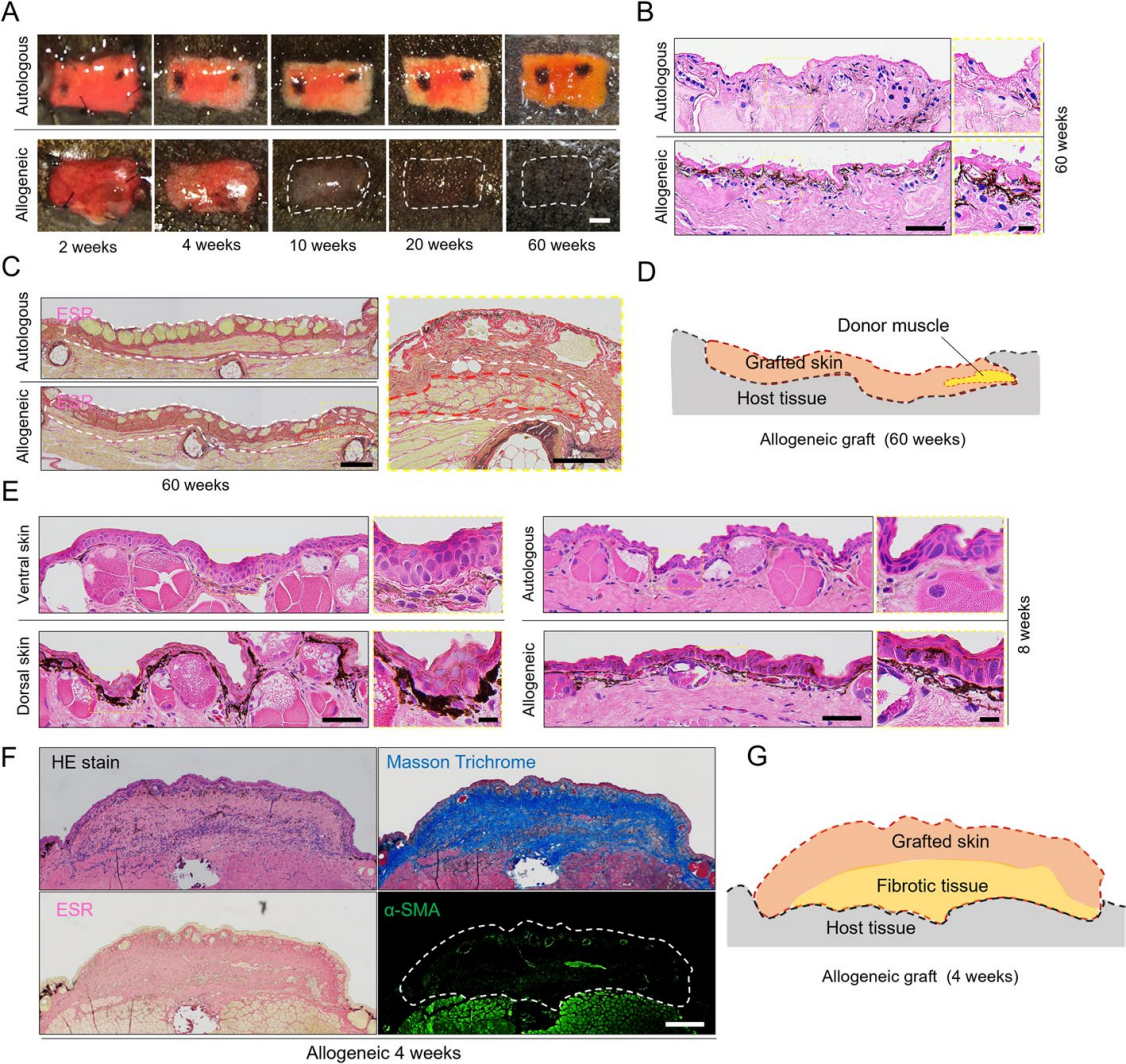
Allogeneic grafts exhibit transient fibrosis with epidermal remodeling and long-term structural integration. **A** Time course of graft appearance. Autologous grafts showed transient whitish discoloration at the margin and regained original ventral coloration by 60 weeks. In contrast, allogeneic grafts lost red donor coloration by 10 weeks, followed by progressive darkening, becoming similar to surrounding dorsal skin by 60 weeks. Dashed lines indicate original graft boundaries. Scale bars, 500 μm. **B** Histological analysis at 60 weeks. A distinct subepidermal pigmented layer was observed in allogeneic but not autologous grafts. Insets show higher-magnification views. This layer corresponds to dorsal skin-like epidermal pigmentation. Scale bars, 100 μm (main) and 20 μm (insets). **C** ESR staining of autologous and allogeneic grafts at 60 weeks. White dashed lines indicate the graft region. In the allogeneic graft, a magnified image of the boxed area is shown on the right. Red dashed lines indicate the boundary of remaining donor muscle, in which muscle fibers were oriented differently from the surrounding host tissue. These findings support the preservation of donor-derived tissue structure. Scale bars, 500 μm in low-magnification images and 200 μm in the magnified image. **D** Schematic of graft structure. Donor skin was transplanted after 90° rotation, resulting in orthogonal alignment between donor and host muscle fibers, enabling identification of donor-derived tissue. The yellow region indicates donor muscle. **E** Epidermal pigmentation after transplantation. Ventral skin lacks pigmentation, whereas dorsal skin contains a dark epidermal layer. At 8 weeks, autologous grafts remained non-pigmented, whereas allogeneic grafts developed dorsal-like pigmentation. Insets show higher-magnification views. Scale bars, 100 μm (main) and 20 μm (insets). **F** Histological characterization at 4 weeks. HE, Masson trichrome, and ESR staining showed marked thickening and collagen accumulation. αSMA staining revealed no αSMA-positive cells, indicating absence of contractile myofibroblasts. Dashed lines indicate the thickened region. Scale bars, 500 μm. **G** Schematic of tissue organization. At 4 weeks after allogeneic transplantation, fibrotic tissue was interposed between grafted skin and host tissue. ESR: Elastica-Sirius Red staining; αSMA: α-smooth muscle actin

Newt dorsal skin contains a melanocyte-rich pigmented layer beneath the epidermis (Fig. 2E), whereas abdominal skin lacks this structure. After long-term observation (60 weeks), the appearance of allogeneic grafts gradually became similar to that of the surrounding dorsal skin (Fig. 2A). Histological analysis demonstrated the formation of a pigmented layer beneath the epidermis in allogeneic grafts (Fig. 2B).

In contrast, autologous grafts retained the original abdominal tissue features, including muscle fibers and mucous glands. In the allogeneic graft, the muscle fibers in the area surrounded by a red dashed line showed an orientation different from that of the underlying host muscle (Fig. 2C). Because the grafts were transplanted with a 90° rotation, muscle fibers oriented differently from the host musculature suggest a distinct tissue origin, supporting the presence of donor-derived tissue structures. At this stage, reactive thickened tissue at the donor–host interface was no longer evident and inflammatory responses had subsided; however, this finding suggests that graft tissue structures were not completely lost (Fig. 2C, D). At 8 weeks after allogeneic transplantation, expansion of the subepidermal pigmented layer beneath the graft was observed, suggesting active remodeling of the epidermal–subepidermal region (Fig. 2E).

To characterize the reactive fibrotic tissue, ESR staining and Masson’s trichrome staining confirmed collagen-rich matrix deposition (Fig. 2F, G). In contrast, αSMA staining was weak and limited, suggesting limited accumulation of contractile myofibroblast-like cells.

These findings demonstrate that allogeneic transplantation induces transient collagen-rich fibrosis that resolves over time in parallel with epidermal remodeling.

### Fibrosis and its regression correspond temporally with leukocyte dynamics

**Fig. 3:**
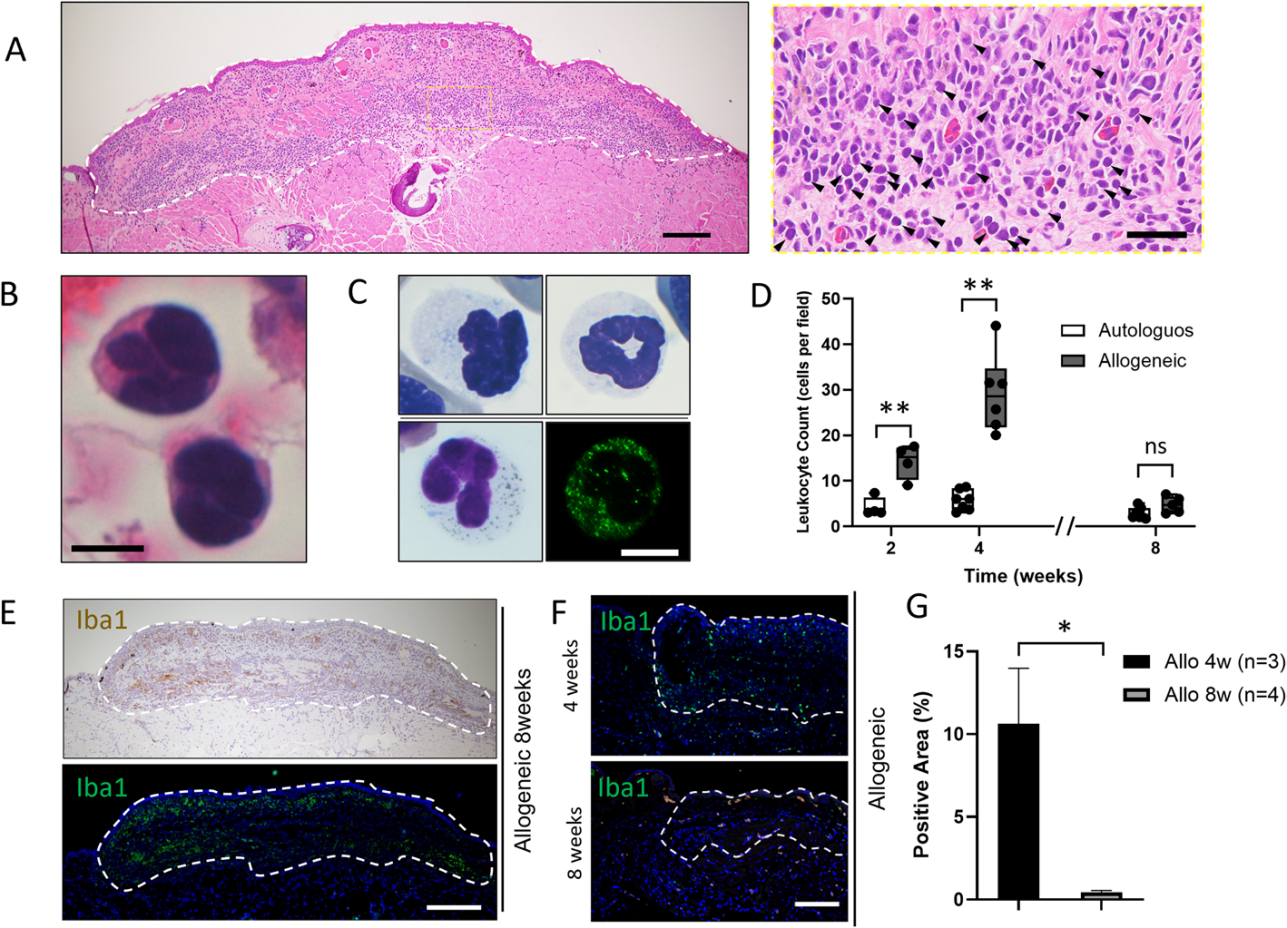
Leukocyte dynamics temporally correlate with fibrotic tissue formation and regression. **A** Histological analysis of allogeneic grafts at 4 weeks post-transplantation showing extensive cellular infiltration within the thickened tissue. The right panel shows a higher-magnification view of the indicated region, with arrowheads indicating infiltrating leukocytes. Scale bars, 500 μm (left) and 100 μm (right). **B** Representative HE-stained image of infiltrating leukocytes within the graft, showing cells with lobulated nuclei. Scale bar, 10 μm. **C** Morphological and functional characterization of infiltrating leukocytes. Upper panels show May–Grünwald–Giemsa–stained cells exhibiting multilobed nuclei consistent with granulocytes. The lower left panel shows a cell following intravenous injection of India ink, with black particles observed within the cytoplasm, indicating phagocytic activity. The lower right panel shows a cell after intravenous injection of DiO-labeled liposomes, demonstrating uptake of fluorescent liposomes, further supporting phagocytic capacity. Scale bar, 10 μm. **D** Quantification of leukocyte numbers within graft tissue. Allogeneic grafts exhibited a marked increase in leukocyte counts at 4 weeks compared to autologous grafts, followed by a decline to near-baseline levels by 8 weeks. **E** Immunohistochemical staining for Iba1 at 4 weeks post-transplantation showing accumulation of macrophages within the grafted area. The upper panels show DAB staining, while the lower panels show immunofluorescence staining. Scale bars, 500 μm. **F** Immunofluorescence staining for Iba1 in allogeneic grafts at 4 and 8 weeks. Iba1-positive cells were abundant at 4 weeks and markedly reduced by 8 weeks. Scale bars, 500 μm. **G** Quantification of Iba1-positive area in allogeneic grafts at 4 and 8 weeks, demonstrating a significant reduction over time. Sample sizes were *n* = 3 (4 weeks) and *n* = 4 (8 weeks). White dashed lines indicate donor boundaries. *p < 0.05, **p < 0.01, ns: not significant. All data are presented as mean ± SEM. DiO: 3,3′-dioctadecyloxacarbocyanine perchlorate; Iba1: ionized calcium-binding adapter molecule 1

At 4 weeks postoperatively, corresponding to the peak fibrotic tissue formation, extensive cellular infiltration was observed within allogeneic grafts (Fig. 3A). Leukocytes with lobulated nuclei were identified based on morphological criteria and validated using peripheral blood smear comparisons (Fig. 3B, C). Because definitive classification for newt leukocyte subsets is not established, cells with lobulated nuclei were classified morphologically as granulocyte-like cells. Quantitative analysis revealed a marked increase in leukocyte numbers in allogeneic grafts compared with autologous controls, peaking at 4 weeks and declining to baseline levels by 8 weeks (Fig. 3D). This temporal pattern paralleled the formation and regression of fibrotic tissue. Focusing on macrophages, Iba1 staining demonstrated robust infiltration within the graft at 4 weeks, whereas Iba1-positive cells were scarcely detected at 8 weeks (Fig. 3E–G). Given that definitive classification of leukocyte subpopulations in newts remains unclear, Iba1-positive cells were regarded as macrophage-lineage cells in this study.

These observations indicate that immune cell dynamics, particularly those of Iba1-positive cells, are temporally associated with fibrosis formation and regression.

### Macrophage depletion delays regression of fibrotic tissue after allogeneic transplantation

To assess the functional role of macrophages during the early phase following allogeneic transplantation, clodronate liposome was used to transiently deplete macrophages prior to transplantation. Effective macrophage depletion in this model was confirmed by a reduction in Iba1-positive cells in the spleen (Supplementary Fig. 1).

**Fig. 4:**
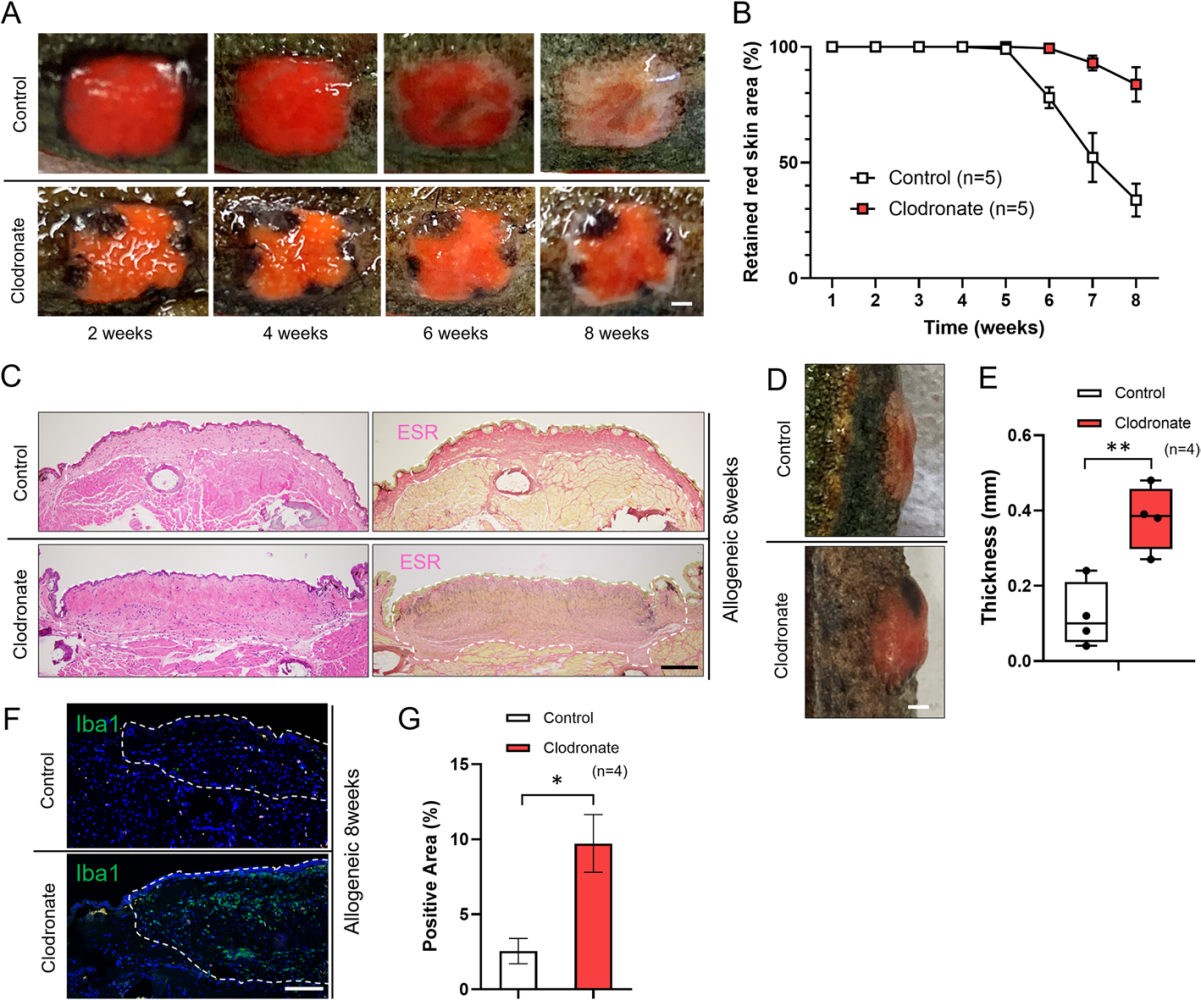
Macrophage depletion delays regression of fibrotic tissue after allogeneic transplantation. **A** Time course of allogeneic graft appearance in control and clodronate-treated animals. While control grafts showed progressive reduction of the red donor area, clodronate-treated grafts retained the donor area. Scale bars, 500 μm. **B** Quantification of retained red skin area over time. Clodronate-treated animals exhibited delayed reduction of the donor-derived area compared with controls. Two-way repeated-measures ANOVA revealed a significant interaction between treatment and time (*p* = 0.0011). **C** Histological analysis at 8 weeks post-transplantation. HE and ESR staining revealed persistent tissue thickening and collagen accumulation in clodronate-treated grafts compared with controls. **D** Macroscopic comparison at 8 weeks post-transplantation. Grafts in clodronate-treated animals displayed persistent elevation and thickening relative to controls. **E** Quantification of tissue thickness at 8 weeks. Fibrotic thickening was significantly increased in clodronate-treated animals compared with controls. **F** Immunofluorescence staining for Iba1 at 8 weeks post-transplantation. Iba1-positive cells were detected within the graft region in the clodronate-treated group, indicating reaccumulation following transient depletion. Dashed lines indicate the graft region. Scale bars, 500 μm. **G** Quantification of Iba1-positive area at 8 weeks showing comparable macrophage presence between groups. Data were analyzed by two-way repeated-measures ANOVA (mixed-effects model, REML), with Welch’s t-tests for individual time-point comparisons. *p < 0.05, **p < 0.01. All data are presented as mean ± SEM. ESR: Elastica-Sirius Red staining; Iba1: ionized calcium-binding adapter molecule 1

Under these conditions, allogeneic grafts were still integrated and developed fibrotic tissue at the donor–host interface, similar to controls. However, while control animals showed resolution of graft thickening and disappearance of the red donor area by 8 weeks, these processes were delayed in clodronate-treated animals (Fig. 4A, B). Fibrotic thickening persisted and was significantly increased at 8 weeks (Fig. 4C–E). ESR staining revealed sustained collagen accumulation, indicating impaired fibrosis regression (Fig. 4C). At 8 weeks after allogeneic grafting, a time point at which swelling had already resolved in control animals, persistent swelling was clearly observed only in the clodronate-treated group, whereas swelling had resolved by this time point in control animals (Fig. 4D). Consistently, thickening of the reactive fibrotic tissue was confirmed in the clodronate-treated group (Fig. 4E). Notably, Iba1-positive cells, initially depleted by clodronate injection, had reaccumulated within the graft by 8 weeks, despite the persistence of fibrotic thickening (Fig. 4F, G).

These findings indicate that macrophages are not essential for the initiation of collagen-rich fibrotic tissue formation but contribute to its timely resolution following allogeneic transplantation.

## Discussion

In this study, skin defects in newts were successfully closed even by allogeneic skin grafting. In contrast to autologous grafts, allogeneic grafts developed a distinct reactive tissue, likely reflecting a fibrotic response associated with immune recognition of non-self tissue. Notably, this fibrosis was transient and progressively regressed over time. While macrophage depletion delayed fibrosis regression, these findings suggest that Iba1-positive macrophage-like (phagocytic) cells may contribute to efficient fibrosis regression.

In humans, damaged skin tissues cannot be fully regenerated, and extensive tissue defects can only be closed gradually from the surrounding tissue [2, 21]. Therefore, for patients with large full-thickness skin defects caused by severe trauma or burns, surgical closure using skin grafting is required [1, 22]. However, allogeneic transplantation elicits strong rejection responses and cannot achieve permanent wound closure. Consequently, autologous skin grafting remains the standard approach in clinical practice [2, 3] but this approach results in fibrotic scar formation, leading to permanent aesthetic and functional impairments.

In contrast to humans, newts have a remarkable capacity for skin regeneration. Previous studies have reported that adult newts have a rapid re-epithelialization capacity, with the wound epidermis covering the wound bed within 1–3 days. Subsequent dermal reconstruction proceeds slowly, and regeneration can be completed over a period of up to 2 years without persistent fibrotic tissue [11]. In the present study, we applied surgical suturing techniques to skin grafting in newts. Autologous grafts achieved stable engraftment without graft loss and maintained their original morphological features over time. Unexpectedly, unlike allogeneic skin grafts in humans, allogeneic grafts in newts were also not shed. Given the rapid re-epithelialization capacity of newt skin, one possible outcome was that epithelialization from the wound edge might separate the non-self graft from the wound bed and lead to graft loss. However, this was not observed. Instead, allogeneic grafts remained attached.

In humans, allogeneic skin grafts are nevertheless used in certain contexts. In skin banking systems, cadaveric skin can be transplanted to temporarily cover tissue defects [23, 24]. However, such grafts are typically rejected within approximately one week due to immune responses and are therefore used only as temporary coverage until autologous grafting can be performed [4, 25]. Interestingly, the temporal course of allogeneic grafts in humans involves an initial phase of transient engraftment with restoration of blood flow within a few days, followed by progressive inflammatory tissue damage and eventual necrosis or sloughing within about one week [25–27]. Notably, this sequence partially overlaps with the phenomena observed in newts. In our model, allogeneic grafts (1) initially showed revascularization (around 2 weeks), (2) subsequently exhibited edematous changes (2–4 weeks), and (3) remained attached without being shed (observed up to 60 weeks). Based on the similarity of phases (1) and (2), we consider that a partial rejection-like response may occur in newts.

In this study, the edematous change observed during phase (2) was shown to represent fibrotic thickening. Specifically, a collagen-rich reactive tissue transiently formed at the interface between the graft and host tissue. In human allogeneic grafts, similar structural changes have been reported to reflect inflammation associated with revascularization [25, 26], suggesting that newts exhibit a comparable initial response. However, the subsequent response diverges markedly: in newts, this fibrotic tissue gradually regressed. This finding suggests that fibrosis can be actively resolved as part of an intrinsic immune response. It also suggests that the allogeneic transplantation model may be a useful system for dissecting mechanisms of fibrosis regression.

In contrast, a major difference was observed in graft fate. In newts, allogeneic grafts were not shed. Instead, they were progressively covered by surrounding pigmented dorsal skin, and by approximately 8 weeks postoperatively, most of the 3 mm graft area was covered. Histologically, this corresponded to the formation of a subepidermal pigmented layer, and the timing of this coverage coincided with fibrosis regression. Although the functional significance remains unclear, it may reflect a remodeling process associated with immune regulation in response to non-self tissue.

Notably, in the longest follow-up (60 weeks), the graft structure was not completely lost. Although this finding suggested retention of graft-associated tissue architecture, it does not directly demonstrate sustained donor-cell viability, functional donor-cell activity, or functional allograft engraftment. Given that extracellular matrix architecture can be preserved even after tissue decellularization [28], it remains possible that such architecture persisted despite loss or replacement of donor cellular components. Therefore, we cannot conclude that non-self donor tissue was functionally engrafted or maintained as viable allogeneic tissue over the long term. Future lineage-tracing or cell-labeling approaches will be required to address this issue.

Fibrosis has traditionally been considered an irreversible end-stage outcome of tissue repair [6]. This is particularly evident in human skin, where extensive skin defects caused by severe trauma or burns result in permanent scar formation and do not fully revert to normal tissue [1, 2, 22, 29]. In contrast, previous studies of amphibian skin wounds have supported the view that regenerative species are able to avoid fibrotic scar formation. In full-thickness skin wounds of both adult Cynops pyrrhogaster and Xenopus, collagen-rich extracellular matrix (ECM) appears, but the wound heals without recognizable scar tissue [11, 17]. However, other studies suggest a different view: regenerative species may also remodel or clear fibrotic-like tissues before maturation. After cardiac cryo-injury, salamander ventricles form a transient collagenous network without compact scar tissue and are fully regenerated by 60–90 days [15]. During normal newt lens regeneration, transient ECM/collagen accumulation is detected at 4 dpl but is cleared by 30 dpl [16]. These observations suggest that regenerative species may have the ability to remodel or resolve fibrotic tissues before they mature into permanent scars. Interestingly, even in humans, in certain organs such as the liver and lung, fibrosis has been shown to be a dynamic and potentially reversible process regulated by specific immune cell populations [7, 30, 31]. Our findings suggest that the regenerative capacity of newts may involve not only avoiding fibrosis but also actively resolving a fibrotic reaction after it has formed.

Previous studies in regenerative amphibians indicated that immune cells, particularly macrophages, are associated with these responses. In an axolotl cardiac cryo-injury model, early macrophage depletion impaired regeneration and resulted in persistent collagen-rich scar-like tissue formation [15]. During newt lens regeneration, macrophage depletion has been reported to inhibit lens regeneration and lead to the formation of scar-like tissue instead of a new lens [16]. In our model, depletion of Iba1-positive macrophage-like cells delayed the regression of collagen-rich fibrotic tissue after allogeneic transplantation. This suggests that Iba1-positive cells are associated with fibrosis regression in newt skin. In this model, clodronate liposome was administered prior to transplantation to transiently deplete macrophages [14], thereby primarily affecting early immune responses to non-self tissue. Notably, collagen-rich fibrotic tissue still formed after depletion, but its subsequent regression was delayed despite the later recruitment of Iba1-positive cells within the graft. These findings suggest that, under our experimental conditions, early Iba1-positive cell responses are not required for the initial formation of collagen-rich fibrotic tissue, but may be important for promoting its subsequent regression.

However, it should be noted that the precise mechanisms remain unclear due to several limitations. First, leukocyte subsets in amphibians are not yet clearly defined at the genetic level, and their conservation across species remains uncertain. Classification in newts and Xenopus has largely relied on morphological criteria [19, 20], which do not fully capture functional or molecular heterogeneity [32]. Even in studies demonstrating the importance of macrophages in regeneration and fibrosis control using clodronate depletion [14, 17], macrophage identity is primarily inferred from antibody staining rather than strict lineage definitions. Similarly, our conclusions regarding macrophages are based on histological staining and depletion experiments. This highlights the need for caution with interpreting immune cell functions in regenerative species. Elucidating the precise classification and functional roles of leukocyte populations in such organisms may provide novel insights into the mechanisms underlying regeneration and fibrosis. Second, the function of the later reappearance of Iba1-positive cells within the graft remains unclear. In an axolotl limb regeneration model, macrophage depletion at a later stage of regeneration did not fully impair the regenerative process [14], suggesting that the importance of macrophage responses may differ depending on the stage of regeneration. Our findings raise the possibility that there is a critical early window during which Iba1-positive macrophage-lineage cell responses promote fibrosis regression. Further studies will be needed to determine how delayed recruitment of Iba1-positive cells influences long-term fibrotic remodeling in newts. Third, it remains unclear what contributes to the regression of collagen-rich fibrotic tissue after allogeneic grafting: regenerative wound-healing mechanisms or mechanisms related to immunological acceptance. The ability of newts to avoid persistent fibrosis [10, 13, 16] may reflect the former, whereas the relatively weak adaptive immune responses of salamanders [13] may reflect the latter. Investigating their relative contributions will be an important next step. Finally, the magnitude of rejection responses is determined not only by major histocompatibility complex (MHC) compatibility [4, 29], but also by additional immunological factors, including minor histocompatibility antigens, cellular immune responses, and humoral immunity [33, 34]. However, in this study, these potential factors were not experimentally evaluated due to the limitations of the experimental system in newts, thus the degree of alloimmune mismatch could not be precisely defined.

The causal relationship between fibrosis and regeneration remains an open question. In clinical settings, keloids—pathological fibrosis—develop more than one month after the onset of wound healing [35]. While fibrosis is often considered a terminal outcome of tissue repair [2], it may also reflect excessive or prolonged repair responses [6, 36]. In our study, fibrosis was observed under conditions of impaired regeneration following macrophage depletion. The ability of newts to resolve fibrosis may therefore provide insight into mechanisms linking fibrosis and regeneration.

## Conclusions

This study demonstrates a unique phenomenon in which allogeneic transplantation in a regenerative organism induces transient fibrosis followed by its resolution. This process suggests the presence of an immune-regulated mechanism that enables the resolution of fibrosis and may contribute to scarless tissue repair after injury.

## Supplementary Information

Supplementary Material 1. Supplementary Fig. 1. Validation of macrophage depletion. (A) Representative images of DiO-labeled liposomes in control and clodronate-treated animals at 3 weeks post-treatment, showing uptake of liposomes. (B) Immunofluorescence staining for Iba1 in spleen sections at 3 weeks demonstrating a marked reduction of Iba1-positive macrophages following clodronate treatment. Scale bars, 20 μm. (C) Quantification of Iba1-positive area in the spleen at 3 weeks, confirming effective depletion of macrophages. *p < 0.05. All data are presented as mean ± SEM. DiO: 3,3′-dioctadecyloxacarbocyanine perchlorate; Iba1: ionized calcium-binding adapter molecule 1.

Supplementary Material 2.

## Data Availability

No datasets were generated or analysed during the current study.

## Abbreviations

PFA: Paraformaldehyde
HE: Hematoxylin and eosin
ESR: Elastica-Sirius Red
DAB: 3,3′-Diaminobenzidine
αSMA: α-Smooth muscle actin
Iba1: Ionized calcium-binding adapter molecule 1
DiO: 3,3′-Dioctadecyloxacarbocyanine perchlorate
SEM: Standard error of the mean
ECM: Extracellular matrix
MHC: Major histocompatibility complex
