# Supplementary figures and images for: Allogeneic skin transplantation induces transient fibrosis that undergoes spontaneous regression in newts
Source: Inflamm Regen. 2026 Aug 1;46:31. doi: 10.1186/s41232-026-00438-0 (PMC13430897; doi:10.1186/s41232-026-00438-0)

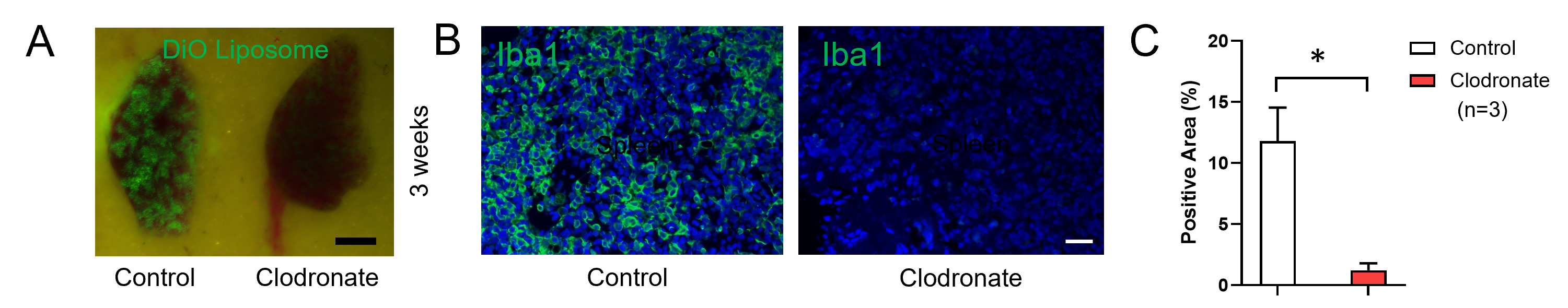

Supplement: Supplementary file 1 — Supplementary Material 1. Supplementary Fig. 1. Validation of macrophage depletion. (A) Representative images of DiO-labeled liposomes in control and clodronate-treated animals at 3 weeks post-treatment, showing uptake of liposomes. (B) Immunofluorescence staining for Iba1 in spleen sections at 3 weeks demonstrating a marked reduction of Iba1-positive macrophages following clodronate treatment. Scale bars, 20 μm. (C) Quantification of Iba1-positive area in the spleen at 3 weeks, confirming effective depletion of macrophages. *p < 0.05. All data are presented as mean ± SEM. DiO: 3,3′-dioctadecyloxacarbocyanine perchlorate; Iba1: ionized calcium-binding adapter molecule 1. [file 41232_2026_438_MOESM1_ESM.tif]
